# Supplementary material for: Urea Cycle Related Amino Acids Measured in Dried Bloodspots Enable Long-Term In Vivo Monitoring and Therapeutic Adjustment
Source: Metabolites. 2019 Nov 12;9(11):275. doi: 10.3390/metabo9110275 (PMC6918381; doi:10.3390/metabo9110275)
Supplement: Supplementary file 1 [file metabolites-09-00275-s001.pdf]

Supplementary:

Supplementary Table 1. Optimised mass spectrometry parameters used for the detection of underivatised urea cycle amino acids in positive ion mode.

Analyte	Retention time (minutes)	Precursor ion (m/z)	Product ion (m/z)	Cone voltage (Volts)	Collision energy (Volts)
L-Arginine	5.79	175.10	69.94	28	20
L-Arginine-13C6	5.79	181.10	73.94	28	20
L-Citrulline	6.60	176.10	69.94	30	18
L-Citrulline-d7	6.60	183.15	76.99	30	18
L-Ornithine	5.73	132.93	69.94	17	17
L-Ornithine-d7	5.73	139.98	76.99	16	15
L-Glutamate	6.57	148.02	83.82	20	11
L-Glutamic acid-d5	6.57	153.06	88.86	20	11
L-Glutamine	6.51	147.06	83.94	18	12
L-Glutamine-13C2	6.51	149.06	84.94	18	12
Argininosuccinate	6.26	291.02	69.87	40	29
ASA anhydrides	6.07	273.12	69.87	40	29

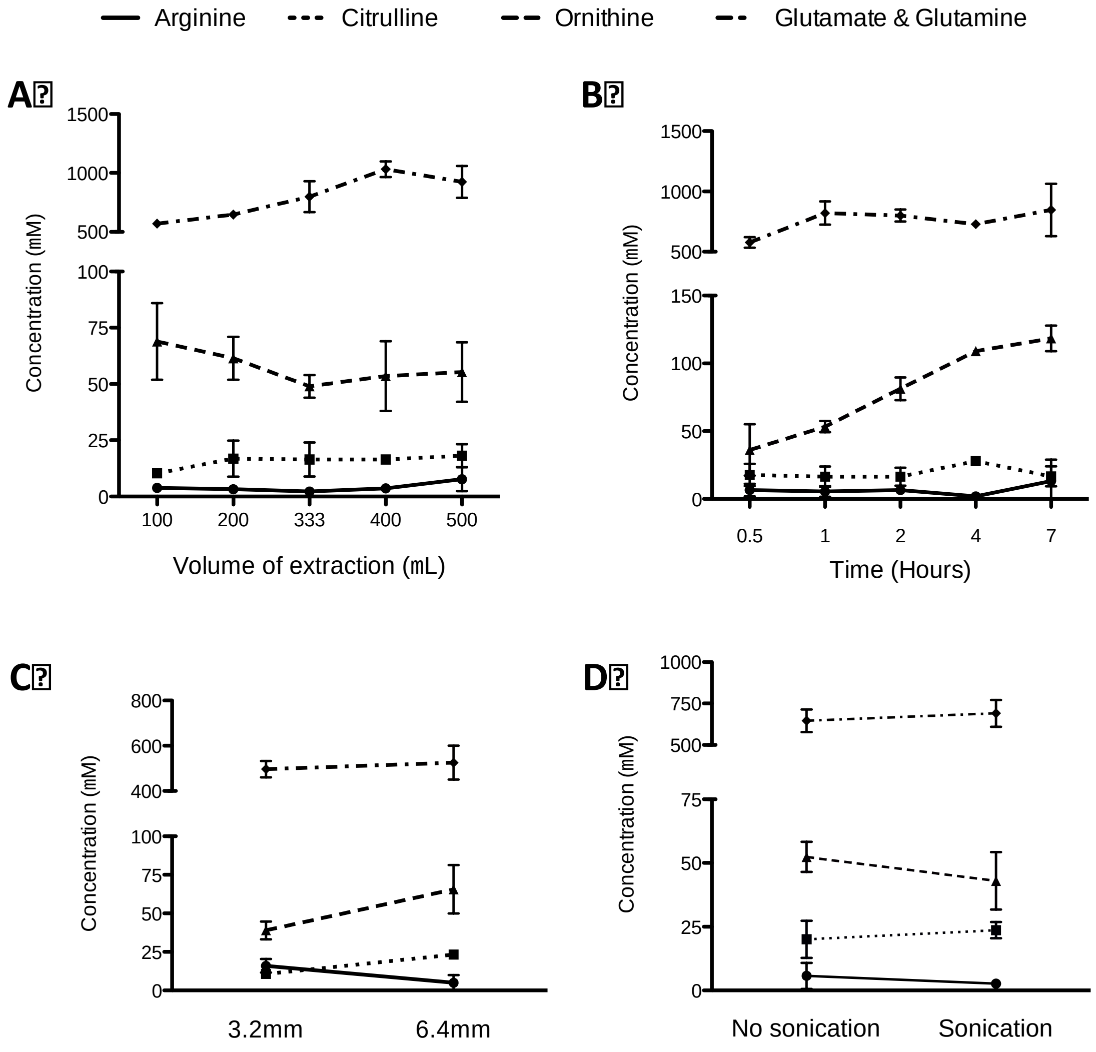

Supplementary Figure 1. Optimisation of dried blood spot extraction. Extraction of L-arginine, L-citrulline, L-ornithine and summed L-glutamine and L-glutamate from dried bloodspots with variation of **(A)** volume of methanol, **(B)** time of elution, **(C)** punch size and **(D)** sonication. Each experiment was performed in triplicates. Graphs represent mean ± standard deviation (SD).

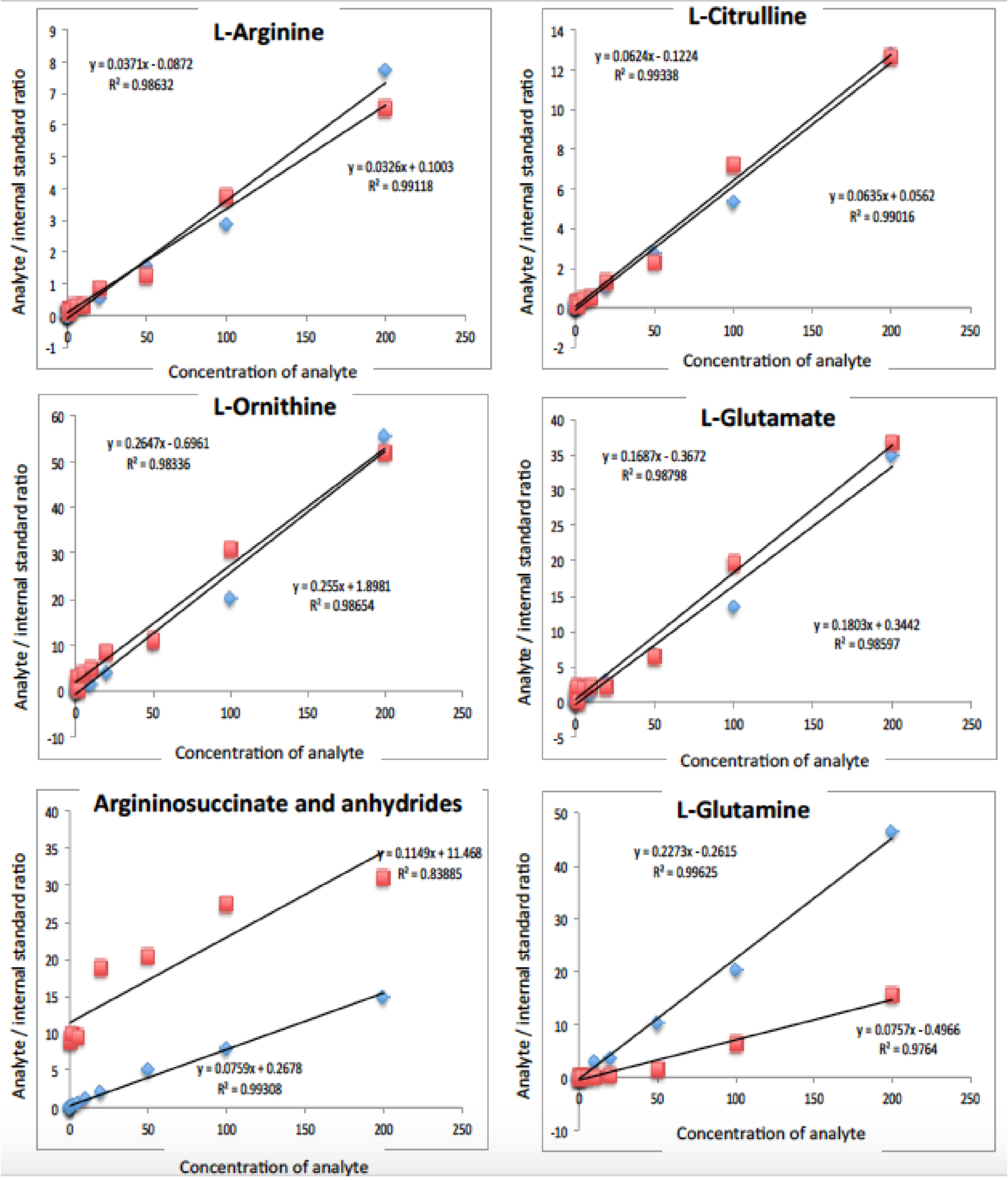

Supplementary Figure 2. Matrix effect. **Study of impact of matrix effect for each analyte.** Standard curves are run in methanol (blue) and spiked in blood then spotted onto a Guthrie card (red), respectively.
